# Atlanta Academy of Medicine

**Published:** 1876-12

**Authors:** J. S. Todd

**Affiliations:** Reporter


### Reports of Societies.

### ATLANTA ACADEMY OF MEDICINE.

J. S. TODD, M.D., REPORTER.

President, Dr. H. V. M. Miller, presiding.

Dr. J. G. Westmoreland has been much troubled of late with a case of lumbago, occurring in a lady naturally unwieldy on account of her size, she weighing nearly two hundred pounds. He desired the views of the Academy in regard to its causation, pathology, and treatment. He had been worried during his professional career in treating it. The usual remedies which he prescribed were blisters or cups to the spine, and quinine internally, but their results were negative.

Dr. W. F. Westmoreland would like to know what lumbago was, and thought that every pain in the back should not be dubbed with this unmeaning word.

Dr. Miller: What do you think it is, Dr. Westmoreland?

Dr. W. F. Westmoreland did not believe in it at all.

Dr. J. G. Westmoreland: What my patient has is acute pain in the lumbar region on the slightest motion of all the members of the body, even the head, the arms alone excepted.

Dr. Miller: Are the pains muscular?

Dr. J. G. Westmoreland: No.

Dr. W. F. Westmoreland: There it is; the muscles are not involved, and still you call it lumbago. It is a term which should be expunged from scientific writings. Liver complaint expresses as much, and is equally as learned an expression. Cystitis, kidney disease, hemorrhoids, and hysteria often cause intense pain in lumbar nerves.

Dr. J. G. Westmoreland: That is all correct, I know, but I want a practical suggestion as to treatment. You may call it a crick in the back, if you will; give me remedies. There is no fever. Remembers to have been very much troubled during the war by a case which lasted for a month, despite all remedies—blistering alone, oft repeated, only bringing temporary relief. Excepting the intense pain caused by motion, no other manifestation of disease was present. Since, however, it is desirable, I will say that my view of the pathology of the disease under consideration does not agree with the idea of lumbago just advanced. The lumbar muscles are not tender or painful in my case, but give intense pain by bending the spinal column in their contraction. The true pathology is, doubtless, that of irritation and engorgement of the meninges, the nerves proceeding from the cord, or the cord itself, and perhaps all of these. The slightest movement which interferes with the quiet of these gives pain.

Dr. J. T. Johnson thought it was a disease *sui generis*, characterized by no pain or other manifestation of disorder, except when there was motion, direct or transmitted to the muscles involved. Has relied for treatment on derivatives and opium principally, hypodermically administered, etc., and cannot say that he has experienced any great embarrassment in its management.

Dr. J. M. Johnson could speak from experience, having suffered from the disease himself. One attack he knows was caused by twitching his back when getting in his buggy, and being caught in the rain just after. He has always regarded it as a severe backache, intensified by sneezing, coughing, or washing his face. Anything that has as a prominent symptom pain in the back, increased by muscular motion, is lumbago. His general health during these spells was as good as ever; he generally cures himself in about five days, by the following remedies: a brisk mercurial purge, turpentine to the back, a hot iron to the back after the turpentine has been applied; hot water he had found grateful, and would blister if these remedies failed.

Dr. W. F. Westmoreland, seven days ago, saw, in consultation, his second case of complete inversion of the womb. The history of the case was not clear, but the attending physician was positive that it was spontaneous. It was nine hours after delivery when he was called. The hemorrhage had been profuse; the vagina still filled with clots; circulation very feeble. We succeeded in reducing it very easily in about ten minutes. His plan of reduction was the same that he used in reducing hernia, namely: with the palm of the hand to the fundus, and fingers grasping the body, he exercised firm compression. This caused the bleeding to recur, until the organ was emptied, when with his fingers (the palm still compressing) he dilated the circular fibres of the os. In a minute and a half the organ had resumed its natural position.

Dr. J. M. Johnson asked if the uterus was passive during his manipulations.

Dr. Westmoreland replied: Yes, except the circular fibres.

Dr. W. F. Westmoreland reported two cases of tracheotomy, both fatal. The first was performed for the removal of a supposed water-melon seed, but found in its stead a thick, hard plug of mucus or fibrine. The child died in ten minutes after the operation. The first cut down to the trachea, and before opening it arrested all hemorrhage, but upon opening the tube a profuse hemorrhage from the mucous membrane, in conjunction with the plug aforesaid, caused a suffocation which was fatal. The child was aged four. The second operation was for the relief of membranous croup, in consultation with Dr. Logan. The obstruction appeared to be confined to the larynx. The child was *in extremis;* pulse not perceptible; breathing almost impossible. The shock caused by the operation was great, but in ten minutes the child had a good pulse, and in a few hours every indication of recovery; even thirty-six hours after the operation the prospects for success were flattering; but about this time it began to suffocate from some obstruction of the tube. Dr. Logan removed the tube, but could not replace it below the obstruction. Thinks we need a new tube in just such cases as this, and is having one made long and floating, that is like a jointed catheter. Has never saved but one case out of fifteen or twenty tracheotomies performed for the relief of membranous croup.

Academy adjourned.

---

Atlanta, Ga., November 7, 1876.

President, Dr. H. V. M. Miller, in the chair.

Dr. J. B. Baird said: Two weeks ago a colored woman came to his office who had been seduced by her employer. Seven days before she had attempted to insert a wire, six inches long, into the womb to produce an abortion. Her efforts to withdraw it were unavailing, hence her visit. She had introduced it about one inch above the meatus. About half inch of the wire was protruding in the vagina. After considerable force the withdrawal was accomplished. It did not enter the womb. He exhibited the instrument which was hook-shaped, and stated that the convexity was towards the womb. She was in the fourth month of pregnancy. He also reported, for the sake of discussion, a case of jaundice in a patient eight years of age. The child had recently had toncilitis which caused some fever. During the attack the patient became yellow; stools clay-colored and semi-solid; urine intensely acid; mind dull; a little pain over the duodenum, *only once*; no tenderness; failed to find any cause for the condition. None of the salts of the bile could be detected in the urine, only the coloring matter. There was a tendency to diarrhœa from first to last. Treatment: sinapisms over the region of the liver—anodyines and alkalies. Duration of case two weeks.

Dr. J. M. Johnson had recently had a very severe case to treat, which lasted one month. First had violent pains in right side; next morning the patient was very yellow; had clay colored, bluish discharges; tenesmus and much pain over duodenum. Calomel, colocynth, hyocyam. and opium in combination, were his main reliance in treating it. She was also blistered. After three weeks treatment, for the first time a copious secretion of bile was set up. During convalescence, to keep the bowels open and clear the skin, hard cider is a favorite remedy with him. Thinks the pathology of the disease will be found to be an inflammation of the duodenum primarily, gradually extending into the ducts. The swelling caused by the inflammation and the altered secretion of tough mucus, together clogging up the outlets to the already secreted bile which is absorbed by the veins.

Dr. Ford agreed with Dr. Johnson in his pathology but said that as it was a known fact, if we inject the bile duct the liquid passes directly to the lymphatics and is emptied by them into the common thoracic and thence into the veins; that he thought this its most probable route into the circulating medium.

Dr. Johnson assented to this view.

Dr. Baird thought an over secretion of bile would also produce jaundice. Sudden jaundice following spasm of the duct could not be explained on this hypothesis, however. The *vis a tergo* being less in the portal circulation also favors absorption, where there is over secretion. He had the pleasure of hearing Dr. Webb, a member of the Alabama Medical Association, read a paper on "Hemorrhagic Malarial Fever," in which he gave a very ingenious theory for the sudden yellowness which is observed in that affection. Chemical examination of the urine failed to detect the salts of the bile in that secretion for two or three days after the skin had become yellow; this being the case, clearly the biliverdin was not the factor which caused it. Hematine, effused in the rete-mucusum, (by derangement of the vaso distensor nerves, or paralysis of the vasor motor,) and these acted on through the thin portion of cuticle, by the oxygen in the air, would, and did in his opinion, cause the sudden bronze hue met with so often and unexpectedly.

Dr. J. G. Westmoreland thinks the pathology of Drs. Johnson, Baird and Ford correct, but differs with Dr. J. in his treatment. His treatment is to counteract the inflammation in the denodenum, for which he blistered and restricted the diet. In entiritis would not give a drastic cathartic, neither would he in duodinitis, nor give acids but alkalis and opiates, and, above all, no mercury; for he does not wish to administer a drug which would causemore bile to be secreted, when that already elaborated failed to find its way into the intestines. Mercury in small doses he considered an irritant or excitant of the alimentary mucous membrane; so of colocynth.

Dr. J. M. Johnson had tried this mode of treatment, and had had much experience. He gave the mercury to lessen inflammation, and if mercury possesses any virtue its chief one is to control inflammation. Results prove everything, and the large bilious secretions following his treatment is all he wants as evidence in favor of his plan. He always blisters. Calomel, colocynth, hyocyam. and opium in combination are soothing and are not drastic or irritating. Thinks the failure on the part of Dr. Baird to find any of the biliary salts in the urine argues against his idea of super secretion. Remarked that if generally the disease is left alone recovery will take place in the large majority of cases, but has seen people die from the disease

*per se.* He does not object to alkalies, but does not consider them curatives.

Dr. J. G. Westmoreland thought that a copious secretion of bile *three weeks* after treatment was poor evidence in favor of Dr. Johnson's plan.

Dr. Baird said that when super secretion was the cause, purgatives would undoubtedly excite a good influence. He gave diuretics in the latter stages of the affection.

Dr. J. G. Westmoreland observed that where there was icterus from over secretion, as was undoubtedly the case in bilious fever, purgatives were indicated to remove impacted feces, and the results justified their use.

Dr. J. M. Johnson thought the glands of Lieberkuhn and Bruner, by being inflamed, secreted a more irritating mucus and in larger quantities, and should be gotten rid of by purgation. Alluded to a case recently under his care which was relieved after emesis of white mucus matter. Purgatives and enemas in huge proportions were unavailing for a week.

Dr. Miller—If the ducts are plugged a sufficient length of time jaundice must necessarily result, but is there an occlusion of the ducts always? Has never read of a post mortem where the patient died of jaundice *per se*, therefore, we have no positive evidence that occlusion always causes it. Saw during the war in three months at least a thousand cases. It was epidemic among the soldiers. He cannot believe that in all these cases there was obstruction, or else there would certainly have been some deaths, which was not the case. How bile gets into the ducts from the cells in the human subject has not, as yet, been discovered. The supposition is that it endosmoses through the attenuated cell membrane. By an alteration of the physical condition of this membrane the process would be interrupted. Inflammations of mucous membranes, when such condition comes immediately under the eye, as sore mouth, for instance, never in his experience caused sufficient swelling to close the duct of Steno; again in inflammation of the bladder, which could be diagnosed with almost absolute certainty, the ureters were not closed. As a rule the ducts of *secreting* glands were not plugged by inflammation of the organs into which they emptied. Thorefore, reasoning from analogy, he would not admit, nor did he believe that duodenitis would always or usually result in a closure of the ductus communis choledocus. He thinks the liver more at fault than the duodenum. Inflammation may be present in the latter occasionally, but as a rule there is no tenderness over it. As to treatment the cases would get well without it, but he thought those who took cider got well the sooner. In obstinate cases where there is constipation he gives purgatives; where the stools were clay colored, mercurials. If he was satisfied there was obstruction of the duct he would give an emetic.

Dr. Connally had used bi-tart. of potas. and was pleased with its effects; thought it closed up the skin and acted on the bowels and kidneys better than any other remedy.

Dr. Baird: Suggested that a catarrh, often caused deafness by closure of the eustachian tube.

Dr. Miller: Said he referred to the ducts from secreting glands, therefore the eustachian tube was not alluded to.

Academy adjourned.

---

ATLANTA, GA., NOV. 21, 1876.

The President, Dr. Miller, in the chair.

Dr. Baird called attention to a very interesting case, which forcibly illustrated the importance of correct diagnosis. The patient was sent to him by a physician for supposed disease of the spinal cord. He had been treated by several other medical men for a supposed affection of the cord, and his symptoms were of a nature to warrant such a deduction, viz.: intense constant pain and weakness in the lumbar region, loss of memory, weakness of the knees, great nervousness; in fact, a general breaking down of the health. Upon close questioning as to the condition of the urinary organs, he further learned that there was frequent emissions of semen, diurnal and nocturnal. Often even the presence of a woman would cause a discharge. It was not always that a complete erection of the penis preceded the seminal discharge. He complained that when he attempted sexual intercourse, the discharge occurred before connection was complete. He had had a gonorrhœa some five or six years previously. An examination of the urethra revealed a stricture, through which with difficulty he introduced a No. 2 bougie. In a short time the dilatation had been so far carried forward that a No. 23 (French) sound was passed. He rapidly improved in every respect; in fact, entirely recovered. As evidence that the constriction of the urethra had caused all these symptoms, he mentioned, that having to leave the city and be absent for two months, he first instructed the patient how to use the sound, but when he returned, for some cause it had been neglected, and the man was fast relapsing into his former condition, which was as promptly corrected by again instituting gradual dilatation. Of all unfortunates this class of patients were the most preyed on by quacks and mountebanks, and his patient was not an exception to the number so fleeced.

Dr. John Thad. Johnson said that he had often had occasion to observe—which at first surprised him—the many reflex nervous phenomena which stricture gave rise to. He made it a rule lately, when obscure pains, referable to the loins, thighs, etc., were complained of, to invariably examine the urethra. It was very easy of exploration—but little more trouble than to inspect the fauces. When the semen is too quickly discharged during intercourse, or there are frequent nocturnal emissions, it denotes a hyperasthesia of the glans penis, which is in the large majority of instances dependent on stricture; frequent urination is also very often caused by it. True stone in the bladder, kidney disease, masturbation, and bad sexual hygiene cause many of these nervous symptoms, but stricture more than the most of us are aware of. An examination of the urethra in most nervous diseases was insisted on by him.

